# Argan Fruit Polyphenols Regulate Lipid Homeostasis, Prevent Liver Fat Accumulation, and Improve Antioxidant Defense in High-Calorie Diet Fed Mice: In Vivo Study and In Silico Prediction of Possible Underlying Mechanisms

**DOI:** 10.3390/metabo15040234

**Published:** 2025-03-28

**Authors:** Mohammadine Moumou, Imane Mokhtari, Mohamed Harnafi, Mohammed Alrugaibah, Thamer Aljutaily, Hend F. Alharbi, Abdulmalik Alhuwaymil, Abdulkarim S. Almutairi, Hassan Barakat, Dragan Milenkovic, Souliman Amrani, Hicham Harnafi

**Affiliations:** 1 Laboratory of Bioresources, Biotechnologies, Ethnopharmacology and Health, Faculty of Sciences, University Mohamed I, Oujda 60 000, Morocco; mohammadine.moumou@ump.ac.ma (M.M.); mokhtari.imane@ump.ac.ma (I.M.); harnafi.mohamed@ump.ac.ma (M.H.); s.amrani@ump.ac.ma (S.A.); h.harnafi@ump.ac.ma (H.H.); 2 Department of Food Science and Human Nutrition, College of Agriculture and Food, Qassim University, Buraydah 51452, Saudi Arabia; m.alrugaibah@qu.edu.sa (M.A.); thamer.aljutaily@qu.edu.sa (T.A.); hf.alharbi@qu.edu.sa (H.F.A.); 451113929@qu.edu.sa (A.A.); 3 Al Rass General Hospital, Qassim Health Cluster, Ministry of Health, King Khalid District, Al Rass 58883, Saudi Arabia; abdulkarimsa@moh.gov.sa; 4 Plants for Human Health Institute, Department of Food, Bioprocessing and Nutrition Sciences, North Carolina State University, Kannapolis, NC 28081, USA; dmilenk@ncsu.edu

**Keywords:** *Argania spinosa*, polyphenols, lipid metabolism, lipid peroxidation, liver steatosis, hypercaloric diet, mice, food supply

## Abstract

**Background/Objectives:** *Argania spinosa* L. Skeels is a Moroccan endemic plant widely used by the local population as folk medicine. This study aimed to investigate the effects of Argan fruit pulp on lipid metabolism disorders and liver steatosis in hypercaloric diet-fed mice. **Methods:** Animals were treated with the Argan fruit pulp extract and its fractions for 12 weeks at 100 and 200 mg Kg^−1^ BW daily. The analysis was conducted on lipid levels in plasma, liver, feces, and bile as well as on glycemia. The liver glutathione, malondialdehyde, and antioxidant enzyme activities were assessed. The hepatic steatosis was evaluated by measuring transaminases and alkaline phosphatase activities and examining histological sections. The polyphenol profiles were determined using HPLC-DAD. Possible underlying mechanisms in the hypolipidemic and hepatoprotective activities were predicted by molecular docking. **Results:** The crude extract and its aqueous fraction (rich in protocatechuic and gallic acids) significantly restored plasma lipids and glucose levels. Indeed, total cholesterol level (TCHO) was decreased in the liver but increased in bile and feces. The treatment also reduced body weight and liver and adipose tissue mass and prevented liver steatosis. The ethyl acetate fraction exhibited no effect on lipid metabolism but significantly prevented liver oxidative stress. The crude extract and its fractions appear to be nontoxic (LD50 > 5000 mg Kg^−1^) in mice. The phenolic acids demonstrated strong binding affinity to key targets involved in regulating lipid homeostasis, including ABCA-1, LXR, CYP7A1, HMH-CoA reductase, and PCSK-9. However, the identified flavonoids exhibited high affinities to targets involved in oxidative stress defense (SOD, CAT, and CYP2E1). **Conclusions:** The Argan fruit pulp, particularly its polyphenols, could be a promising natural approach for preventing cardio-metabolic diseases by improving lipid metabolism and reducing liver oxidative stress.

## 1. Introduction

*Argania spinosa* (L.) Skeels (locally named Argan tree), a member of the *Sapotaceae* family, is a native species found mainly in southwest Morocco and in some mountainous regions as a wild crop in the country’s east [1]. In the mountainous areas of southern Morocco, the Argan tree represents an essential component of the regional ecology, cultural legacy, and traditional practices [2]. Local populations have used different parts of the Argan tree for culinary, cosmetic, and medicinal purposes [2]. Indeed, the fruit seeds were predominantly used to extract the Argan oil, which is widely known for its nutritional and cosmetic properties [3]. However, the other parts of the tree, such as leaves, fruit pulp, and kernel, have long been used as a traditional medicine to treat several diseases, such as diabetes mellitus, hyperlipidemia, rheumatism, dry skin, and gastritis [4].

The pulp is the main byproduct of the artisanal preparation of Argan oil, constituting up to 40% of the entire fruit [5]. The pulp is rich in phenolic compounds, mainly phenolic acids and flavonoids such as protocatechuic acid, gallic acid, quercetin, luteolin, hesperidin, hyperoside, and isoquercetin [6]. A recent study showed the presence of newly isolated compounds in the pulp of Argan grown in Spain, including vaccihein A, mirificin, bergapten, avicularin, and prodelphinidin B_4_ [5]. Among many other polyphenols, these compounds were suggested to benefit cardio-metabolic health [7,8]. Indeed, Martínez and coworkers have demonstrated that the polyphenol-rich extract from Spanish Argan pulp positively affected glucose metabolism, inflammation, and oxidative stress in a mouse model [5]. Furthermore, Hebi et al. [9] highlighted the hypoglycemic effect of South Moroccan Argan fruit pulp in diabetic rats. It is important to note that a few studies have focused on the pharmacological effect of Argan oil from eastern Morocco [10,11]. Still, no study has yet been carried out to highlight the phytochemical composition of local Argan fruit pulp and its possible beneficial effects against lipid metabolism disorders and related diseases.

Although some studies have been conducted on the pharmacological properties of *Argania spinosa*, they mainly focused on its oil and leaves, with limited data available on the fruit pulp. Azizi et al. [12] comprehensively reviewed the phytochemistry and pharmacological activities of different parts of the Argan tree, highlighting its antioxidant, antidiabetic, and anti-inflammatory properties. A detailed phytochemical analysis of Argan fruit pulp (collected in Chtouka ait Baha, south of Morocco) conducted by El Monfalouti et al. revealed the presence of various polyphenolic compounds, including catechin, epicatechin, procyanidin B1, procyanidin B2, epigallocatechin gallate, isoquercitrin, hyperoside, rutin, phloridzin, myricetin, quercitrin, and quercetin [13]. The study also demonstrated that Argan pulp exhibits intense antioxidant activity, which could contribute to its potential health benefits in metabolic disorders. These findings support the need for further investigation into the pharmacological properties of Argan fruit pulp, particularly its effects on lipid metabolism.

Hyperlipidemia and oxidative stress are key contributors to the progression of non-alcoholic fatty liver disease (NAFLD), atherosclerosis, and cerebrovascular diseases, which represent leading causes of mortality in both Western and Eastern populations [14]. These pathologies are generally due to consuming high-calorie diets rich in fats and sugars [15]. Actually, a positive correlation has been established between the consumption of the “Western diet” and the occurrence of several metabolic disorders, including insulin resistance, hyperlipidemia, NAFLD, and other metabolic/cardiovascular diseases [16].

Interestingly, this study aimed to investigate the effects of a crude extract from East Moroccan Argan fruit pulp and its aqueous and organic fractions on lipid disorders, hepatic antioxidative defense, and liver steatosis in mice fed a hypercaloric diet. Specifically, the key objectives were the elucidation of the phenolic HPLC-DAD profiles and content of the pulp extract and its fractions; the investigation of the effect on lipid metabolism, obesity, liver antioxidant defense, and liver steatosis; as well as the comprehension of the possible pathway by which lipid homeostasis was restored.

The originality of this work stems from the fact that the Argan tree, native to the eastern region of Morocco, has not been extensively studied through experimental means. In particular, the fruit pulp’s chemical composition and pharmacological properties have yet to be investigated.

## 2. Materials and Methods

### 2.1. Chemicals and Drugs

Cholesterol, gallic acid, protocatechuic acid, catechin, epicatechin, rutin, hyperoside, quercetin, luteolin, trichloroacetic acid, thiobarbituric acid, butylated hydroxyanisole, aluminum chloride, β-carotene, triton WR-1339, hematoxylin, eosin, polyvinylpyrrolidone, Folin–Ciocalteu reagent, and all the solvents were of analytical grade and purchased from Merck (MERCK, Erstein, France). The fenofibrate drug was purchased from SOTHEMA Laboratories, Casablanca, Morocco. 

### 2.2. Preparation of the Argan Pulp Extract (AAPE) and Its Fractions

#### 2.2.1. Preparation of the AAPE

*Argania spinosa* was identified by a botanist, and a voucher specimen was deposited under the collection number A.S.09. The plant’s name was checked with the World Flora Online database. The fruits at the maturity stage were collected in “Jebel Tikarmin”, belonging to the rural commune of Chouihia (East Morocco, 35°4′47″ N, 3°15′0″ W, altitude 1200 m). The pulps were removed, dried in an air-circulating oven at 40 °C, and ground into fine powder. The dried powder was extracted once in boiling water (100 g 500 mL^−1^) under sonication for 30 min (Elma, Singen, Germany; sonication amplitude of 25 kHz and maximum power of 100 W). The supernatant was filtered and dried at 40 °C to obtain a dry powder from the crude aqueous extraction.

#### 2.2.2. Fractionation of the AAPE by Liquid-Liquid Extraction

The AAPE (10 g) was dissolved in 100 mL distilled water, filtered, and defatted thrice with n-hexane (100 mL) to remove chlorophylls and fats. The aqueous extract was then partitioned three times with ethyl acetate (150 mL) using a separating funnel. The aqueous (AF) and ethyl acetate (EF) fractions were concentrated using a rotary evaporator under reduced pressure and then dried in an air-circulating oven at 40 °C and stored at 4 °C until further use. The extraction yields, expressed as a percentage of the initial dried crude extract, were determined using the following formula:

Yield of extraction (%) = (mass of fraction/mass of crude extract) × 100

### 2.3. Determination of Total Polyphenols, Flavonoids, and Total Tannin Contents

#### 2.3.1. Total Polyphenol Content of the AAPE and Its Fractions

The total polyphenol content was determined using the Folin–Ciocalteu method [17]. In this procedure, 0.5 mL of AAPE (1 mg mL^−1^ in distilled water), AF (1 mg mL^−1^ in distilled water), or EF (1 mg mL^−1^ in ethanol) was combined with 0.25 mL of Folin–Ciocalteu reagent and allowed to react for 5 min. Then, 0.5 mL of saturated sodium carbonate solution was added, and the mixture was incubated at room temperature for 30 min. After incubation, the absorbance was measured at 725 nm. The polyphenol content was quantified using a catechin standard solution calibration curve and expressed in mg/g of dry extract or fraction.

#### 2.3.2. Flavonoids Content of the AAPE and Its Fractions

Total flavonoid content was quantified as previously described by Harnafi et al. [17]. An aliquot of 0.5 mL of AAPE (1 mg mL^−1^ dissolved in distilled water), AF (1 mg mL^−1^ dissolved in distilled water), or EF (1 mg mL^−1^ dissolved in ethanol) was mixed with 1 mL of the aluminum chloride reagent. The mixture was left to develop a yellow color for 30 min, after which the absorbance was measured at 430 nm. The flavonoid amount was calculated using a rutin standard solution calibration curve and expressed in mg g^−1^ of dry extract or fraction.

#### 2.3.3. Total Tannins Content of the AAPE and Its Fractions

Tannins were determined after their complexation with polyvinylpyrrolidone (PVP), following the method outlined by Harnafi et al. [17]. Thus, 5 mL of each extract or fraction at 200 mg mL^−1^ (AAPE and FA dissolved in distilled water and EA dissolved in ethanol) was mixed with 500 mg of PVP under stirring for 30 min. The complex tannin–PVP was allowed to develop for 24 h at 4 °C. After centrifugation at 4000 rpm for 15 min, the non-tannin phenolic compounds in the supernatant were measured using the Folin–Ciocalteu method, as previously described. The tannin concentration was then calculated by subtracting the non-tannin content from the total polyphenol content. The amount of tannin was expressed in mg catechin per gram of dry extract or fraction.

### 2.4. HPLC-DAD Analysis of the AAPE and Its Fractions

The HPLC analyses were carried out using an HPLC-DAD system Agilent 1100 series (Agilent Technologies, Santa Clara, CA, USA) as previously reported [18]. Briefly, 10 µL of the AAPE or its fractions (dissolved in methanol) were filtered through a 0.45 µm filter and injected into a C18 reversed-phase column (250 × 4.6 mm, particle size of 5 µm, Waters Symmetry, USA). The separation was undertaken using a gradient of acidified ultrapure water (A) and methanol (B) at a flow rate of 1 mL min^−1^. A gradient elution mode was set as follows: 0–20 min: 20% B; 20–35 min: 100% B; 35–40 min: 20% B. Phenolic compounds were detected at 280 nm, and the compounds were identified based on their retention times and UV–visible spectra in comparison with a database of standard phenolic compounds. The amount of each phenolic compound (hyperoside, catechin, epicatechin, rutin, quercetin, luteolin, gallic acid, and protocatechuic acid) was determined using calibrated curves of standards.

### 2.5. Animals and Treatments

#### 2.5.1. Preparation of the Hypercaloric Diet (HCD)

The HCD was prepared in the laboratory following our previously established method, with slight modifications [19]. A standard mice diet, obtained from “Provimac-Morocco” (containing 17% proteins, 2% lipids, 57% carbohydrates, 10% minerals, 14% cellulose, and an energy value of 3186.6 kcal/kg), was supplemented with beef fat (16%), cholesterol (1.5%), egg yolk (10%), sucrose (10%), and deoxycholic acid (0.2%).

#### 2.5.2. Experimental Schedule

Adult male albino mice weighing 24–26 g were bred in the Faculty of Sciences, University Mohamed I, Oujda, Morocco animal facility. The mice were given unrestricted access to both food and water throughout the experimental period. Their housing was maintained at a temperature of 22 °C ± 1 with a 12 h light–dark cycle. The animals were treated according to the Directive 2010/63/EU, 2010 guidelines of laboratory use [20] and approved by the institutional committee for using laboratory animals (Approval Number: AS.032023). All measures were taken during handling and treatment to avoid animal suffering.

The experimental mice were divided into six groups of eight animals in individual cages. The normal control group (NCG) was fed a standard diet and gavaged with distilled water. The hyperlipidemic control group (HCG) was fed the HCD and given distilled water. The low-dose AAPE-treated group (AAPEG-100) was fed the HCD and gavaged with the AAPE at 100 mg Kg^−1^ BW. The high-dose AAPE-treated group (AAPEG-200) was fed the HCD and gavaged with the AAPE at 200 mg Kg^−1^ BW. The aqueous fraction-treated group (AFG) was fed the HCD and gavaged with the aqueous fraction at 100 mg Kg^−1^ BW. The ethyl acetate fraction-treated group (EFG) was fed the HCD and gavaged with the ethyl acetate fraction at 100 mg Kg^−1^ BW. The fenofibrate-treated group (FEG) was fed the HCD and gavaged with fenofibrate at 4 mg Kg^−1^ BW similarly.

Food intake for each animal was recorded daily, and body weight was recorded weekly. After 6 and 12 weeks, blood samples were taken from the retro-orbital sinus in tubes containing sodium citrate as an anticoagulant. The samples were immediately centrifuged (2500 rpm/10 min), and plasma was used for biochemical analyses. At the end of the experiment (12 weeks), the animals were sacrificed by cervical dislocation; liver and adipose tissue were removed, washed in saline, and weighed.

#### 2.5.3. Plasma Biochemical Analyses

Triglycerides (TGs), total cholesterol (TCHO), LDL cholesterol (LDL-c), HDL cholesterol (HDL-c), glucose (Glu), alanine aminotransferase (ALT), aspartate aminotransferase (AST), and alkaline phosphatase (ALP) were determined using appropriate biomedical kits (Biosystems kits, Barcelona, Spain).

#### 2.5.4. Liver, Fecal, and Biliary Lipid Analysis

Following the lipid extraction process, as described by Moumou et al. [21], TCHO and TGs in the liver and feces were measured using enzymatic assays [19]. Biliary cholesterol was quantified with an enzymatic kit (Biosystems kits, Barcelona, Spain) after bile was collected from the gall bladder using a 30-gauge needle.

#### 2.5.5. Measurement of Hepatic Oxidative Stress

The extent of oxidative stress in mouse livers was assessed by evaluating reduced glutathione (GSH) levels, malondialdehyde (MDA) content, and the enzymatic activities of superoxide dismutase (SOD) and catalase (CAT). MDA was quantified spectrophotometrically based on its reaction with thiobarbituric acid, following the method of Mokhtari et al. [19]. GSH levels were measured following its reaction with 5,5-dithiobis-(2-nitrobenzoic acid) [22]. The activities of the liver enzymes SOD and CAT were determined using previously established methods [23,24].

#### 2.5.6. Histology of the Liver

Small sections of fresh liver from all treated mice were fixed in 10% buffered formalin, embedded in paraffin, and then sectioned and stained with hematoxylin and eosin. The histological slides were examined under an optical microscope [25].

### 2.6. Study of the Anti-Lipoprotein-Rich Plasma Oxidation In Vitro

The in vitro oxidation of plasma lipoproteins was induced by copper sulfate (CuSO_4_). The oxidative process was followed by measuring the production of thiobarbituric acid reactive substances (TBARSs), as reported by Touiss et al. [26]. Briefly, lipoprotein-rich plasma was recovered from mice treated with Triton WR-1339 (an inhibitor of lipoprotein lipase) at 400 mg Kg^−1^ for 24 h. The LDL-c concentration of the used plasma was 403.22 ± 18.12 mg dL^−1^.

The preventive effect of AAPE, AF, and EF against copper-induced lipoprotein oxidation was studied as follows:

- Normal control: lipoprotein-rich plasma incubated with normal saline only;
- Oxidized control: lipoprotein-rich plasma incubated with CuSO_4_ solution;
- AAPE: lipoprotein-rich plasma incubated with CuSO_4_ and AAPE at increasing concentrations (2–200 µg mL^−1^);
- AF: lipoprotein-rich plasma incubated with CuSO_4_ and AF at increasing concentrations (2–200 µg mL^−1^);
- EF: lipoprotein-rich plasma incubated with CuSO_4_ and EF at increasing concentrations (2–200 µg mL^−1^);
- BHA: lipoprotein-rich plasma incubated with CuSO_4_ and BHA (standard antioxidant) at increasing concentrations (2–200 µg mL^−1^).

### 2.7. Measurement of Anti-Lipoperoxyl Radical Activity

The capacity to scavenge free lipoperoxyl radicals produced during linoleic acid peroxidation was estimated as the percentage inhibition of oxidative β-carotene blanching in vitro. This experiment was conducted according to the method outlined by Benchagra et al. [27], with slight modification. Firstly, an emulsion of linoleic acid and β-carotene was prepared as follows: 2 mg of each compound was dissolved in 1 mL of chloroform in the presence of 200 mg of Tween 40. Then, after the evaporation of chloroform, 100 mL of distilled water was added to the residue. Secondly, 950 µL of the emulsion was added to 50 µL of AAPE, AF, EF, or BHA at different concentrations (2–200 µg mL^−1^) and incubated for 24 h. The absorbance of the blank and samples was recorded at 470 nm. The anti-lipoperoxyl radical activity, which is proportional to the intensity of the maintained color of β-carotene, was calculated as follows:

% Inhibition = 100 − [(Blank*_Absorbance_* − Sample*_Absorbance_*/Blank*_Absorbance_*) × 100]

### 2.8. Oral Acute Toxicity of the AAPE and Its Fractions

The acute toxicity evaluation of the AAPE and its two fractions was carried out according to the protocol described in OECD Guideline 425 [28]. Adult albino mice of both sexes, weighing 27–30 g, were fasted overnight and assigned to groups of four animals (two male and two female) housed individually. Each group was then gavaged with the AAPE, AF, or EF at increasing doses (500, 1000, 2000, and 5000 mg/Kg). The animals were observed for mortality and other morphologic symptoms of toxicity over 2 weeks.

### 2.9. Molecular Docking Analysis

The in silico study was performed using the SwissDock version: 2023 program.

(https://www.swissdock.ch/docking (accessed on 10 November, 2023)). Protein 3D structures were obtained from the UniProt Data Bank (https://www.uniprot.org (accessed on 10 November 2023)), and chemical structures of metabolites from the PubChem database (https://pubchem.ncbi.nlm.nih.gov (accessed on 10 November 2023)). The binding interactions were studied for the following targets: ABCA-1 (ATP-binding cassette receptor) and LXR (liver X receptor), involved in the cholesterol reverse transport pathway; CYP7A1 (cholesterol 7-alpha hydroxylase) and FXR (Farnesoid X receptor), implicated in bile acid synthesis; HMG-CoA-R reductase (Hydroxymethyl glutaryl CoA reductase), which is involved in cholesterol biosynthesis; PCSK9 (proprotein convertase subtilisin/kexin type 9) engaged in LDL-c catabolism; SOD and CAT, implicated in antioxidant defense; CYP2E1 (cytochrome P450 7A1), implicated in oxidative stress; and NAFLD. The binding affinities were expressed in kcal/mol, with values lower than −7 kcal/mol suggesting potential interactions.

### 2.10. Statistical Analysis

Data were analyzed using the Student t-test and one-way analysis of variance (ANOVA). (The normality of the data distribution was assessed using the Shapiro–Wilk test, and the homogeneity of variances was verified using Levene’s test. Tukey’s post hoc test was applied for pairwise comparisons to determine specific differences between groups. The differences with *p*-values less than 0.05 were considered statistically significant. The results are expressed as mean ± SE.

## 3. Results

### 3.1. Phenolic Composition of the AAPE and Its Fractions

#### 3.1.1. Total Polyphenols, Tannins, and Flavonoids Content

Analysis of the major phenolic classes in AAPE showed high amounts of total phenols, with a level of 403.12 ± 2.22 mg g^−1^, 220.65 ± 2.37 mg g^−1^ in total flavonoids and 90.71 ± 1.96 mg g^−1^ in tannins. After fractionation of AAPE between water and ethyl acetate, phenolic classes in the obtained fractions were assayed again. The results showed that the ethyl acetate fraction (EF) was significantly higher in flavonoids than the aqueous fraction (AF), with a level of 195.88 ± 1.54 mg g^−1^ compared with 18.04 ± 1.11 mg g^−1^ (*p* < 0.001). However, the AF appears to be richer in tannins than the EF. The tannin content of the AF was 40.3 ± 1.54 mg g^−1^, while the EF contained only 5.4 ± 1.03 mg g^−1^ (*p* < 0.001) (Table 1).

#### 3.1.2. HPLC Analysis

**Figure 1:**
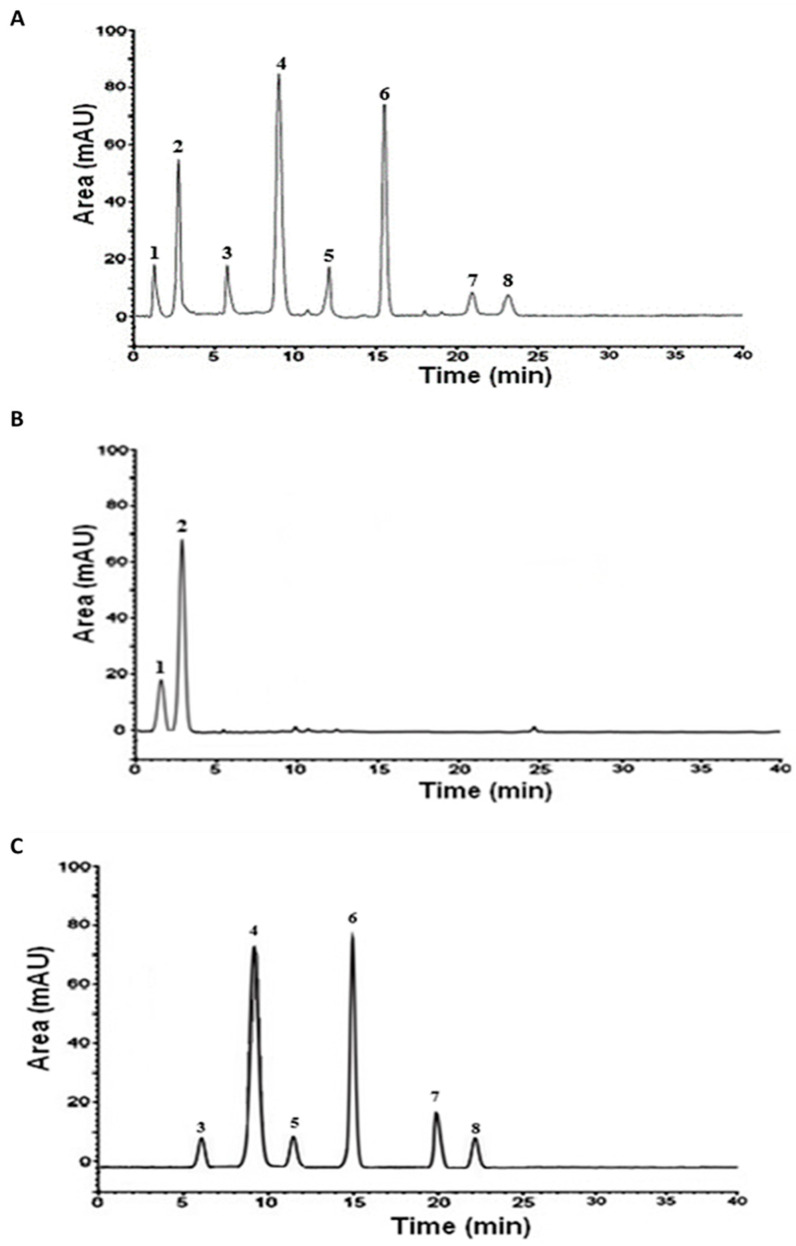
HPLC analysis of the aqueous Argan extract (AAPE) and its fractions. (**A**) AAPE; (**B**) aqueous fraction; (**C**) ethyl acetate fraction. 1: gallic acid; 2: protocatechuic acid; 3: catechin; 4: epicatechin; 5: rutin; 6: hyperoside; 7: quercetin; 8: luteolin.

The HPLC analysis showed that the crude extract contained a mixture of flavonoids and phenolic acids, including hyperoside, catechin, epicatechin, rutin, quercetin, luteolin, gallic acid, and protocatechuic acid. However, fractionation enabled these compounds to be separated according to their respective polarities. The aqueous fraction appeared to be rich in phenolic acids, predominated by protocatechuic acid, while the ethyl acetate fraction contained flavonoids with epicatechin as the predominant (Table 2 and Figure 1).

### 3.2. Metabolic Disturbances Induced by the High-Calorie Diet in Mice

**Figure 2:**
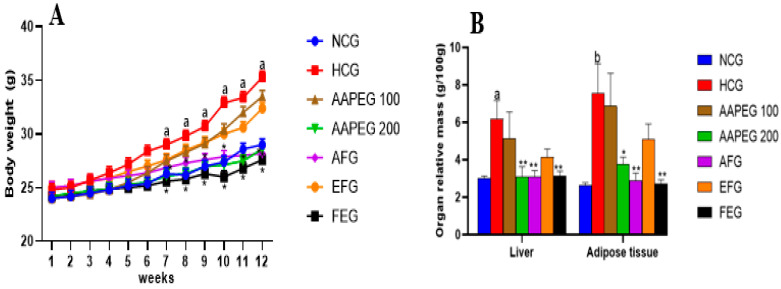
Effect of Argan fruit pulp extract and its fractions on mice body weight (**A**) and organ relative mass (**B**). NCG: normolipidemic control group; HCG: hyperlipidemic control group; AAPEG: Argan aqueous pulp extract-treated group; AFG: aqueous fraction-treated group; EFG: ethyl acetate fraction-treated group; FEG: fenofibrate-treated group. The results are expressed as mean ± SE (*n* = 8). ^a^ *p* < 0.05 and ^b^ *p* < 0.01 vs. NCG. * *p* < 0.05, ** *p* < 0.01 vs. HCG.

The ingestion of the hypercaloric diet (HCD) for a period of 12 weeks caused several metabolic and cellular changes in the mice. Body weight, abdominal adipose tissue mass, and relative liver weight were significantly increased in the HCD group compared with the control group (Figure 2).

**Figure 3:**
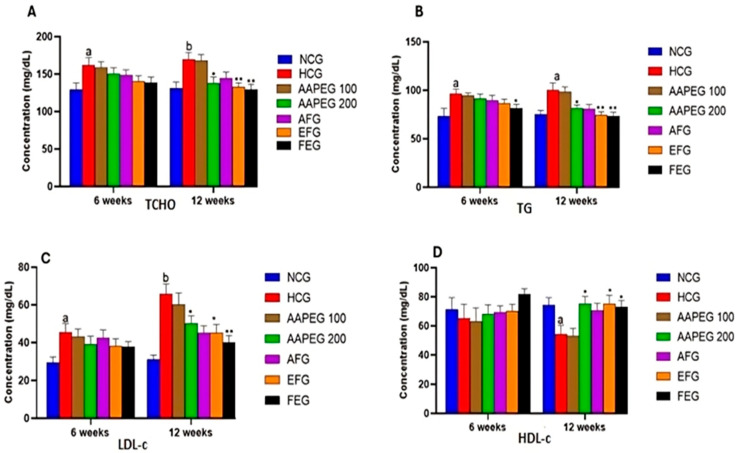
Effect of Argan fruit pulp extract and its fractions on mice plasma lipid profiles: TCHO (**A**), TGs (**B**), LDL-c (**C**), and HDL-c (**D**). TCHO: total cholesterol; TGs: triglycerides; HDL-c: high-density lipoprotein cholesterol; LDL-c: low-density lipoprotein cholesterol. NCG: normolipidemic control group; HCG: hyperlipidemic control group; AAPEG: Argan aqueous pulp extract-treated group; AFG: aqueous fraction-treated group; EFG: ethyl acetate fraction-treated group; FEG: fenofibrate-treated group. The results are expressed as mean ± SE (*n* = 8). ^a^ *p* < 0.05 and ^b^ *p* < 0.01 vs. NCG. * *p* < 0.05, ** *p* < 0.01 vs. HCG.

**Figure 4:**
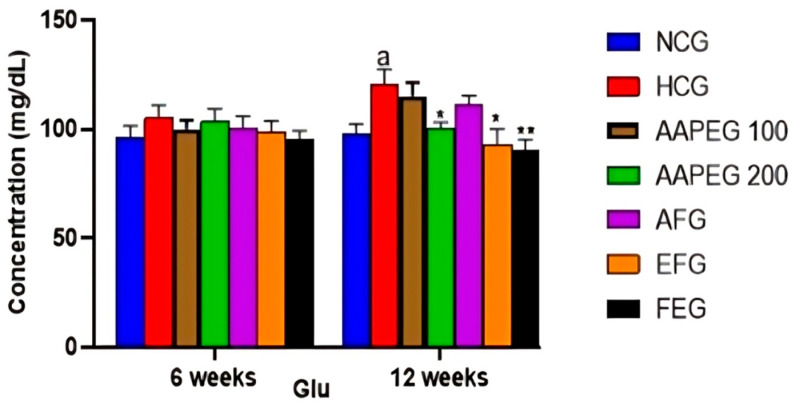
Effect of Argan fruit pulp extract and its fractions on mice plasma glucose. NCG: normolipidemic control group; HCG: hyperlipidemic control group; AAPEG: Argan aqueous pulp extract-treated group; AFG: aqueous fraction-treated group; EFG: ethyl acetate fraction-treated group; FEG: fenofibrate-treated group. The results are expressed as mean ± SE (*n* = 8). ^a^ *p* < 0.05 vs. NCG. * *p* < 0.05, ** *p* < 0.01 vs. HCG.

**Figure 5:**
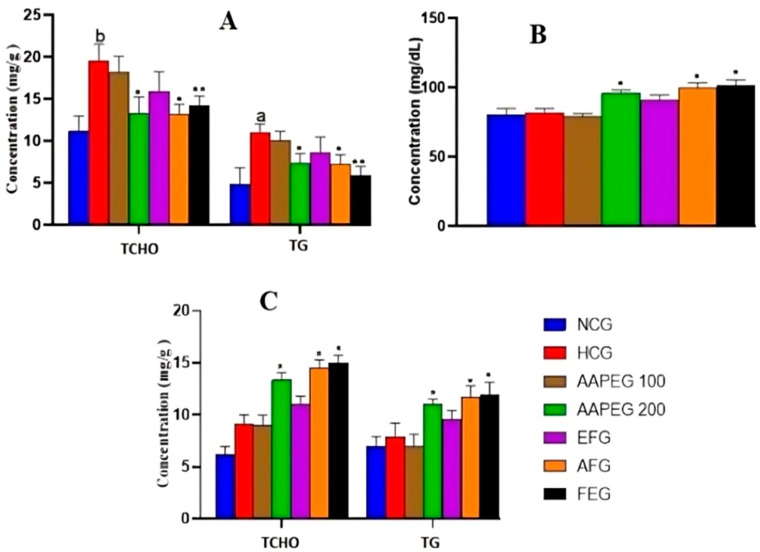
Effect of Argan fruit pulp extract and its fractions on hepatic lipid levels (**A**), biliary TCHO (**B**), and fecal lipids excretion (**C**) in mice. TCHO: total cholesterol; TG: triglyceride; NCG: normolipidemic control group; HCG: hyperlipidemic control group; AAPEG: Argan aqueous pulp extract-treated group; AFG: aqueous fraction-treated group; EFG: ethyl acetate fraction-treated group; FEG: fenofibrate-treated group. The results are expressed as mean ± SE (*n* = 8). ^a^ *p* < 0.05 and ^b^ *p* < 0.01 vs. NCG. * *p* < 0.05, ** *p* < 0.01 vs. HCG.

**Figure 6:**
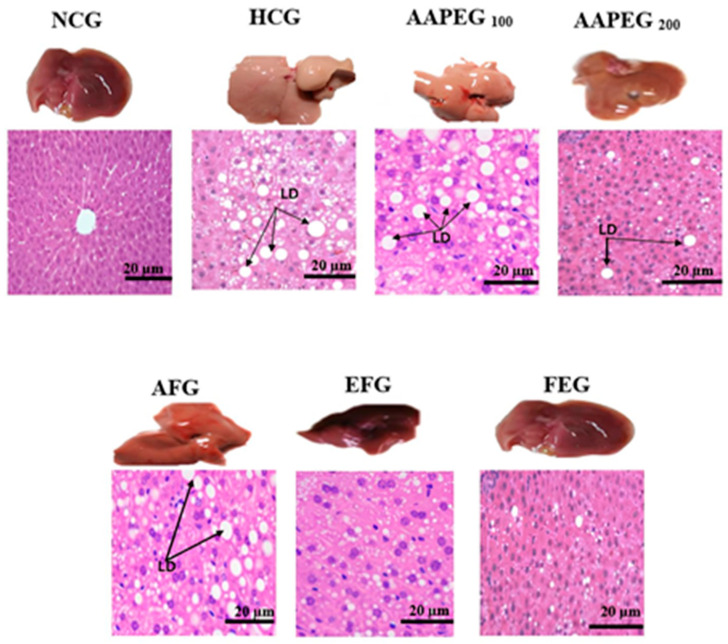
Effect of Argan fruit pulp extract and its fractions on liver morphology and histology. LD: lipid droplets; NCG: normolipidemic control group; HCG: hyperlipidemic control group; AAPEG: Argan aqueous pulp extract-treated group; AFG: aqueous fraction-treated group; EFG: ethyl acetate fraction-treated group; FEG: fenofibrate-treated group.

The intake of this diet also resulted in a disturbance of the lipid and glucose metabolisms, especially after 12 weeks’ treatment, manifested by a significant increase in TCHO (29%, *p* < 0.01), TGs (33%, *p* < 0.05), LDL-c (116%, *p* < 0.01), and Glu (22%, *p* < 0.05) and a decrease in HDL-c (27%, *p* < 0.05) (Figure 3 and Figure 4). This had repercussions on the structure and metabolism of the liver, showing that the liver steatotic environment was beginning to take. The TCHO and TGs in the liver were increased by 75% (*p* < 0.01) and 26% (*p* < 0.05), respectively (Figure 5). Furthermore, the color of the livers of the HCD-fed mice appeared whitish with a fatty appearance, indicating the excessive accumulation of lipids (Figure 6). This was accompanied by an increase in plasma enzymes of liver injury, including AST (+125%, *p* < 0.001), ALT (+30%, *p* < 0.01), and ALP (+14%, *p* < 0.01) (Figure 8). The AST/ALT ratio was also increased (+88%, *p* < 0.001), which indicates liver steatosis.

### 3.3. Metabolic Restoration Exerted by the AAPE and Its Fractions in Comparison with Fenofibrate

As noted, the AAPE at a low dose of 100 mg Kg^−1^ BW exerted no significant metabolic changes in HCD-fed mice. So, all the effects presented below are relative to the dose of 200 mg Kg^−1^.

#### 3.3.1. Effect on Food Intake, Body Weight, Relative Liver Weight, and Abdominal Fat Accumulation

Daily monitoring of food intake showed no significant differences between all the groups, indicating that neither the hypercaloric diet nor the Argan extracts affected the mice’s appetite. After 12 weeks of treatment, it appeared clear that the hypercaloric diet caused weight gain in the mice. This gain became significant only after the 7th week when the weight increased by 10% (*p* < 0.05), and at the end of the treatment, there was a 22% weight gain (*p* < 0.05). In the group treated with low-dose AAPE and its ethyl acetate fraction, the shape of the curve was almost identical to that of the hyperlipidemic group, which means that these two treatments did not significantly impact weight gain. However, AAPE at high dose, its aqueous fraction, and fenofibrate, respectively, reduced weight gain by 18% (*p* < 0.05), 20% (*p* < 0.05), and 22% at the end of the experiment (Figure 2A).

The hypercaloric diet also hindered the relative weight of the liver and abdominal adipose tissue (Figure 2B). In fact, after 12 weeks, liver mass increased by 104% (*p* < 0.05) and abdominal adipose tissue by 186% (*p* < 0.01). However, the AAPE at high dose, AF, and fenofibrate reduced the relative liver weight by 49% (*p* < 0.01), 51% (*p* < 0.01), and 49% (*p* < 0.01), respectively. The loss of adipose tissue mass was 50% (*p* < 0.05), 62% (*p* < 0.01), and 64% (*p* < 0.01), respectively, with the same treatments. The other treatments had no significant effect (Figure 2B).

#### 3.3.2. Effect on Plasma Lipid and Glucose

Based on the obtained results, it is clear that neither the crude extract nor its aqueous and organic fractions significantly affected the plasma lipid parameters after 6 weeks of treatment. However, after 12 weeks, except for the aqueous fraction, the crude extract and its ethyl acetate fraction had statistically significant effects.

Indeed, as seen in Figure 3 and Figure 4, the AAEP at 200 mg Kg^−1^ reduced TC by 18% (*p* < 0.05), LDL-c by 23% (*p* < 0.05), TGs by 19% (*p* < 0.05), and Glu by 16% (*p* < 0.05), while increasing HDL-c by 38% (*p* < 0.05). The effect of the AF fraction was slightly more remarkable than that of the crude extract but was statistically comparable. Indeed, TC was reduced by 22% (*p* < 0.01), LDL-c by 31% (*p* < 0.05), TG by 26% (*p* < 0.01), and Glu by 22.5% (*p* < 0.05), with an increase in HDL-c of 38% (*p* < 0.05). After 6 weeks, fenofibrate appeared to reduce only TGs by 15% (*p* < 0.05). However, after 12 weeks, the effect concerned all parameters. TCHO decreased by 23% (*p* < 0.01), LDL-c by 38% (*p* < 0.01), TGs by 27% (*p* < 0.01) and Glu by 25% (*p* < 0.01), while HDL-c increased by 37% (*p* < 0.05) (Figure 3 and Figure 4). It is essential to point out that no statistical difference was noted between the effect of fenofibrate and those of AAPE and FA for all the parameters measured (*p* > 0.05) (Figure 3 and Figure 4).

#### 3.3.3. Effect on Hepatic, Biliary, and Fecal Lipids

Concerning the effect on hepatic lipids, it can be seen that AAPE and its aqueous fraction are always the most active, while FE remains without any remarkable effect. Indeed, the AAPE significantly reduced TCHO and TGs concentration in the liver, with scores of 31% (*p* < 0.05) and 30% (*p* < 0.05), respectively (Figure 5A). The AF reduced the same parameters by 31.5% (*p* < 0.05) and 36% (*p* < 0.05), respectively. Also, fenofibrate reduced hepatic TCHO by 26% (*p* < 0.01) and TGs by 46% (*p* < 0.01). In this respect, the effect of the drug remained statistically comparable to that of AAPE and AF (*p* > 0.05) (Figure 5A). The beneficial metabolic effects of Argan fruit pulp extracts were also clearly observed in biliary cholesterol (Figure 5B). Indeed, compared with the hyperlipidemic group, the AAPE increased biliary cholesterol by 17% (*p* < 0.01), while the AF increased it by 22% (*p* < 0.01). Fenofibrate had an effect of 24% (*p* < 0.01), which was still not statistically different from the effects of the extracts (*p* > 0.05) (Figure 5B).

The measurement of the TCHO and TGs excreted in feces showed that the AAPE, AF, and fenofibrate had a significant elimination effect (Figure 5C). Thus, the AAPE eliminated 44% (*p* < 0.05) of TCHO and 39% (*p* < 0.05) of TGs. The AF eliminated 59% (*p* < 0.05) of TCHO and 48% (*p* < 0.05) of TGs, while fenofibrate eliminated these two lipids with scores of 62% (*p* < 0.01) and 33% (*p* < 0.05), respectively (Figure 5C).

### 3.4. Effect of the AAPE, Its Fractions, and Fenofibrate on Hepatic Steatosis and Oxidative Stress Markers

#### 3.4.1. Hepatic Tissue Histology and Morphology

Morphological examination showed that the liver in the HCG group was hypertrophic, pale, and deformed compared with the NCG group, which exhibited a uniform reddish-brown color and a normal ovoid shape. After treatment, a dose-dependent improvement was observed: AAPE at 100 mg/kg slightly alleviated these alterations, while the extract at 200 mg/kg further reduced lipid accumulation, though some abnormalities persisted. AF showed moderate recovery, whereas EF and fenofibrate almost completely restored the normal liver appearance. Histological analysis confirmed these trends: HCG exhibited extensive lipid droplet accumulation, while AAPE at 100 mg/kg partially reduced this overload, with a more pronounced decrease at 200 mg/kg. AF showed an improvement similar to AAPE, at 200 mg/kg, but less significant than EF and fenofibrate, where the hepatic structure was nearly restored, with very few or no lipid droplets. Thus, EF and fenofibrate are the most effective at normalizing liver morphology and histology (Figure 6).

#### 3.4.2. Hepatic MDA, Glutathione, SOD, and Catalase

**Figure 7:**
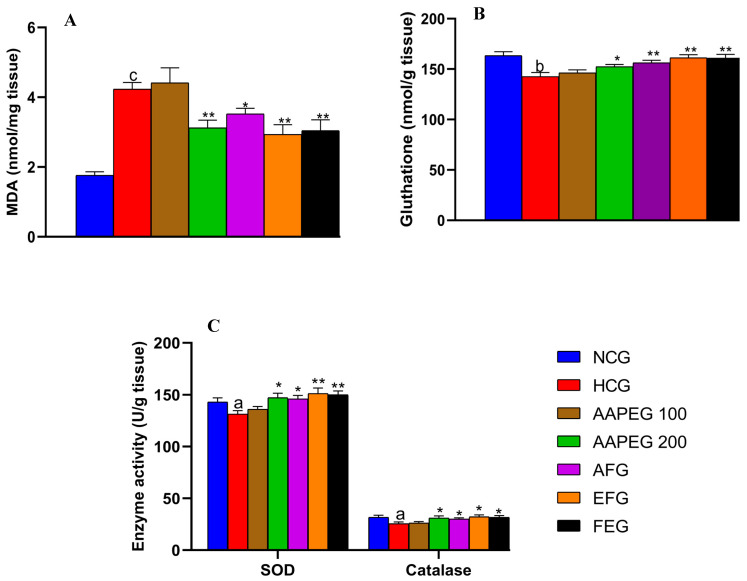
Effect of Argan fruit pulp extract and its fractions on liver oxidative stress markers. (**A**): MDA; (**B**): reduced glutathione; and (**C**): SOD and CAT. MDA, malondialdehyde; SOD, superoxide dismutase; CAT, catalase; NCG, normal control group; HCG, hyperlipidemic control group; AAPEG, aqueous Argan pulp extract-treated group; AFG, aqueous fraction-treated group; EFG, ethyl acetate fraction-treated group; FEG, fenofibrate-treated group. ^a^ *p* < 0.05, ^b^ *p* < 0.01, and ^c^ *p* < 0.001 vs. NCG. * *p* < 0.05, ** *p* < 0.001 vs. HCG.

As seen in Figure 7, the hypercaloric diet exerted a significant increase in the liver MDA content (+140%, *p* < 0.001) accompanied by a reduction in glutathione levels (−11%, *p* < 0.01) and the activity of the enzymes SOD (−8%, *p* < 0.05) and catalase (−19%, *p* < 0.05). In contrast, a higher dose of AAPE resulted in a 20% decrease in hepatic MDA content (*p* < 0.01) (Figure 7A), along with a 12% increase in SOD activity (*p* < 0.05) and a 25% increase in catalase activity (*p* < 0.05) (Figure 7C).

After fractionation, the activity was maintained in both fractions. The EF appeared more active than the aqueous fraction and the crude extract (*p* < 0.01). Obviously, AF reduced MDA by 16% (*p* < 0.05), while EF reduced it by 30% (*p* < 0.01) and fenofibrate by 28% (*p* < 0.01) (Figure 7A). Liver glutathione content (Figure 7B) was also restored by AAPE (+7%, *p* < 0.05), AF (+10%, *p* < 0.01), EF (+13%, *p* < 0.01), and fenofibrate (+12%, *p* < 0.01).

As for SOD (Figure 7C), it appears that AF increased its enzymatic activity by 11% (*p* < 0.05), while EF increased it by 15% (*p* < 0.01) and fenofibrate by 8% (*p* < 0.05). We observed also an enhancing effect on catalase activity exerted by AF (12%, *p* < 0.05), EF (18%, *p* < 0.05), and fenofibrate (15%, *p* < 0.05).

#### 3.4.3. Effect on Transaminases and Alkaline Phosphatase Enzymes

**Figure 8:**
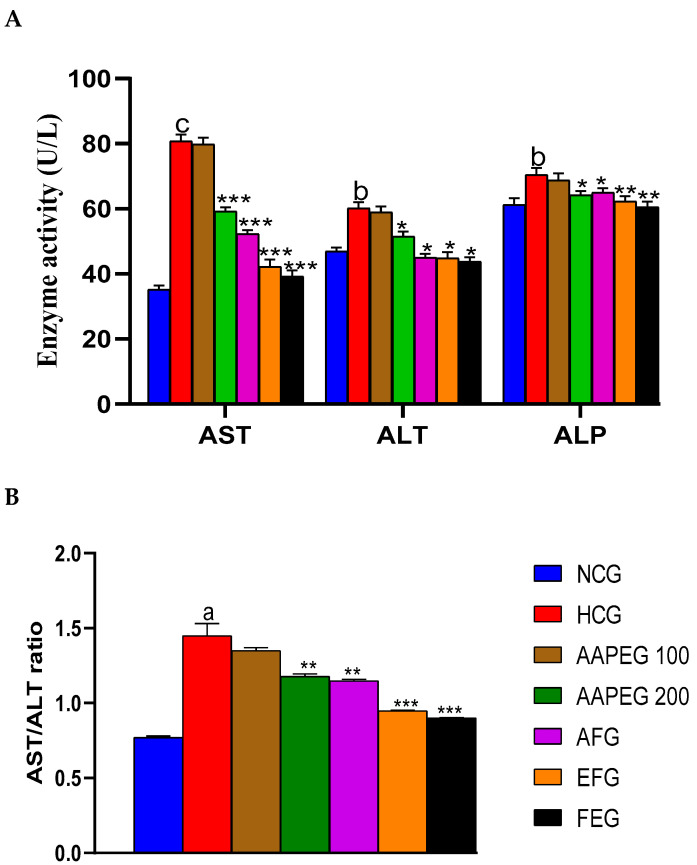
Effect of Argan fruit pulp extract and its fractions on mice’s transaminase and phosphatase alkaline activities. (**A**): ALT, AST, and ALP; (**B**): AST/ALT ratio. AST: aspartate aminotransferase; ALT: alanine aminotransferase; ALP: alkaline phosphatase; NCG, normal control group, HCG, hyperlipidemic control group; AAPEG, aqueous Argan pulp extract-treated group; AFG, aqueous fraction-treated group; EFG, ethyl acetate fraction-treated group; FEG, fenofibrate-treated group. ^a^ *p* < 0.05, ^b^ *p* < 0.01, and ^c^ *p* < 0.001 vs. NCG. * *p* < 0.05, ** *p* < 0.01, *** *p* < 0.001 vs. HCG.

As mentioned above, the hypercaloric diet significantly increased the level of AST, ALT, the AST/ALT ratio, and alkaline phosphatase, indicating hyperlipidemia-related steatosis. However, these increases were significantly suppressed in the treated groups (Figure 8A,B). Actually, AST activity was reduced by 26% (*p* < 0.001), 35% (*p* < 0.001), 47% (*p* < 0.001), and 51% (*p* < 0.001) in groups treated with AAPE, AF, EF, and fenofibrate, respectively. As for ALT activity, it was decreased by 15% (*p* < 0.05), 25% (*p* < 0.05), 26% (*p* < 0.05), and 28% (*p* < 0.05), respectively, in the same treated groups (Figure 8A). This resulted in a significant restoration of the AST/ALT ratio, which appears to be decreased by 18% (*p* < 0.05), 20% (*p* < 0.05), 34% (*p* < 0.05), and 37% (*p* < 0.05), respectively, in the same groups cited above (Figure 8B). Alkaline phosphatase activity was also lowered in the treated groups compared with the control by, respectively, 8% (*p* < 0.05), 7% (*p* < 0.05), 11% (*p* < 0.01), and 14% (*p* < 0.01) in AAPEG, AFG, EFG, and FEG (Figure 8A).

### 3.5. In Vitro Inhibition of Plasma Lipid Peroxidation and Anti-Lipoperoxyle Radical Scavenging Activity

**Figure 9:**
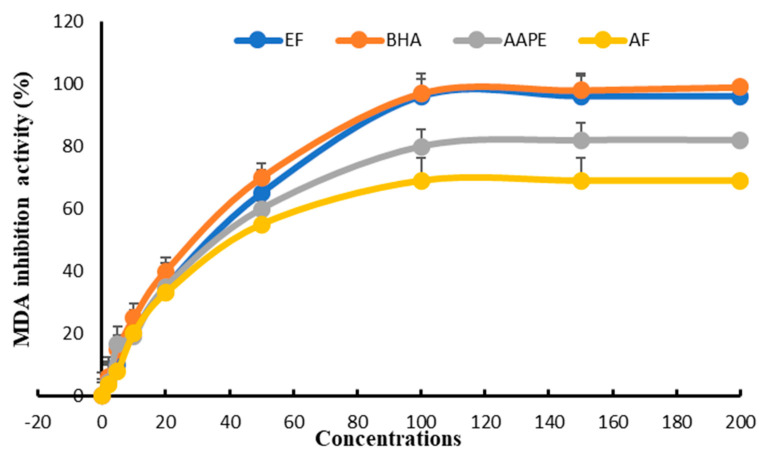
Effect of Argan fruit pulp extract and its fractions on plasma lipid oxidation. AAPE: aqueous Argan pulp extract; EF: ethyl acetate fraction; BHA: butylated hydroxyanisole; AF: aqueous fraction.

The study of inhibiting lipoprotein-rich plasma peroxidation in vitro enabled us to classify the three extracts according to their IC_50_ compared with BHA. EF appeared to be the most active, with an IC_50_ = 27.33 ± 0.61 µg mL^−1^ compared with 25.81 ± 0.52 µg mL^−1^ for BHA. In second place was AAPE, with an IC_50_ = 40.12 ± 0.38 µg mL^−1^, and in last place was AF, with an IC_50_ = 46 ± 0.78 µg mL^−1^ (Figure 9).

**Figure 10:**
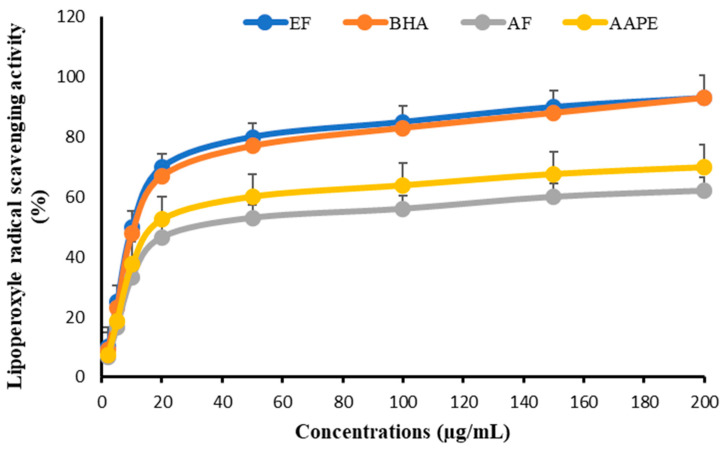
Effect of Argan fruit pulp extract and its fractions on lipoperoxyle radical scavenging. AAPE: aqueous Argan pulp extract; EF: ethyl acetate fraction; BHA: butylated hydroxyanisole; AF: aqueous fraction.

As for the lipoperoxyl anti-radical effect, the same pattern of curves was observed. The impact of EF was the highest, with an IC_50_ of 11.57 ± 0.56 µg mL^−1^, which was comparable to that of BHA at 11.78 ± 0.83 µg mL^−1^. AAPE had an IC_50_ value of 18.84 ± 0.44 µg mL^−1^ and an AF value of 20.13 ± 0.62 µg mL^−1^ (Figure 10).

### 3.6. In Silico Interaction of Argan Fruit Pulp Phenolic Compounds with Key Proteins Involved in Lipid Metabolism and Oxidative Stress

Table 3 summarizes the binding forces, measured in kcal mol^−1^, between various polyphenols from Argan pulp and key proteins involved in the regulation of lipid metabolism and oxidative stress. As principal results, the data reveal that protocatechuic acid (PCA) and gallic acid (GA) exhibit strong binding interactions with multiple proteins, highlighting their potential as effective modulators of lipid metabolism pathways. For example, PCA stands out for its consistently strong binding across most proteins, particularly with the liver X receptor (LXR) and proprotein convertase subtilisin/kexin type 9 (PCSK-9), where it achieves binding forces of −10.15 and −10.11 kcal mol^−1^, respectively. This suggests that PCA could significantly influence lipid homeostasis by interacting with these critical regulators. GA also demonstrates potent binding, particularly with cholesterol 7-alpha hydroxylase (CYP7A1) and PCSK-9, with binding forces of −9.91 kcal/mol and −9.89 kcal/mol, respectively. This indicates that GA could play a key role in modulating cholesterol metabolism.

Catechin (CA), epicatechin (ECA), rutin (RUT), hyporoside (HYP), quercetin (QUE), and luteolin (LUT) show strong binding affinities across superoxide dismutase 2 (SOD2) and catalase (CAT), key enzymes involved in the defense against oxidative stress. Despite their moderate impact on lipid metabolism, these flavonoids may significantly enhance antioxidant defenses and help mitigate oxidative damage.

Furthermore, RUT and HYP exhibit strong binding interactions with cytochrome P450 2E1 (CYP2E1), reflecting their potential impact on the enzyme’s activity and its role in oxidative stress. Specifically, among the polyphenols analyzed, RUT exhibits the highest binding affinity (−10.12 kcal/mol) to CYP2E1. This strong binding suggests that RUT could effectively inhibit or modulate CYP2E1, thereby potentially reducing the production of reactive oxygen species and mitigating oxidative damage.

The data suggest that phenolic acids found in Argan fruit pulp, particularly PCA and GA, could effectively modulate essential lipid metabolism pathways. While having moderate effects on lipid regulation, flavonoids may contribute significantly to the antioxidant defense system, highlighting their potential role in preventing oxidative damage and related diseases.

### 3.7. Acute Toxicity

The acute toxicity study revealed no toxicity signs were observed in animals treated with doses ranging from 500 to 5000 mg/kg BW during the 14-day observation period. This indicates that AAPE and its two fractions can be considered nontoxic, with an LD_50_ value exceeding 5000 mg/kg BW.

## 4. Discussion

The regular consumption of hypercaloric diets rich in fat and sugar is known to be linked to the development of most metabolic and cardiovascular diseases, especially obesity, diabetes, hyperlipidemia, non-alcoholic fatty liver disease (NAFLD), hypertension, and atherosclerosis [15].

To treat hyperlipidemia and reduce cardiovascular risk, many populations around the world have turned to natural treatments based on ethno-medicinal wisdom [29]. In fact, in Morocco, as in many other countries, people resorted to traditional medicine, generally based on treatment with endemic medicinal plants and specific ethnic practices [30]. Among the medicinal plants endemic to Morocco, the Argan tree has long been considered a traditional treatment for cardiovascular disorders [4]. However, few preclinical or clinical studies are available on this subject to date. In this study, we sought to highlight any possible beneficial metabolic effects of Argan fruit pulps in mice fed a high-calorie diet (HCD) for three months.

All around, we noticed that the HCD disrupted several metabolic parameters at the plasma, liver, biliary, and fecal levels. This resulted in overweight, accumulation of abdominal and liver fat, hyperlipidemia, and hyperglycemia, in addition to disturbances of transaminases and hepatic antioxidant enzymes (SOD and CAT). Adding to this, the decrease in hepatic glutathione, the increase in MDA levels, and the morphological and histological changes in the liver can only indicate the establishment of NAFLD-related metabolic syndrome, as mentioned in several previous studies [16,21,31].

Treatment with Argan fruit pulp extracts also alleviated metabolic problems caused by the hypercaloric diet. To elucidate the mechanisms, we explore the significant metabolic effects by examining the potential role of the identified polyphenols in regulating disrupted metabolic pathways. This is achieved by correlating the in vivo study results with in silico protein–ligand interactions and mechanisms referenced in the literature.

Interestingly, regarding lipid metabolism, the crude aqueous extract and its aqueous fraction reduce high levels of TCHO while increasing its HDL fraction, which ensures reverse cholesterol transport (RCT) from peripheral tissues to the liver. This suggests that the polar phenolic compounds in Argan fruit pulp may activate the return pathway of cholesterol metabolism. As shown in vivo, Argan extracts reduced levels of liver cholesterol while promoting its concentration in the bile, facilitating excretion through the intestine and feces. Several investigations have postulated that this mechanism can treat metabolic diseases with phytochemicals, especially phenolic ones [32,33]. It is now recognized that activation of the RCT pathway activates ATP-binding cassette A-1 transporters (ABCA-1), which are directly controlled by liver X receptors and purify cholesterol from peripheral tissues to the liver [34]. Based on the results of molecular docking, the phenolic compounds of the polar fraction (protocatechuic acid and gallic acid) show a strong capacity to bind to ABCA-1 and the LXR receptor, which could explain the effect of the aqueous fraction in maintaining cholesterol homeostasis via the RCT pathway. In addition, this could be confirmed by the fact that the two compounds of the aqueous fraction bind strongly to the CYP7A1 enzyme and its nuclear regulator, Farnesoid X receptor (FXR), which are responsible for converting cholesterol into bile acids, as proposed in other reports on phenolic compounds [7].

On the other hand, the attachment of these phenolic acids to the LXR could partly explain the enhancement of direct cholesterol excretion from the intestine into the feces via the trans-intestinal cholesterol efflux mechanism. Moreover, cholesterol homeostasis may also be enhanced by suppressing hepatic endogenous biosynthesis, mediated by the HMG-CoA reductase enzyme, as previously reported for phenolic compounds [35]. In the same vein, we noted that the two phenolic acids present in the aqueous fraction also bind strongly to the HMG-CoA reductase enzyme, suggesting their probable effect in inhibiting this enzyme and thus reducing the de novo synthesis, contributing to the maintenance of normal cholesterol levels. Several reports have highlighted phenolic compounds’ involvement in regulating this enzyme’s activity [36]. More importantly, the AAPE and its aqueous fraction modulated plasma TCHO and significantly reduced LDL atherogenic cholesterol, indicating that the phytochemicals in these extracts could activate peripheral tissues and liver LDL cholesterol uptake via its receptor B/E, as shown in other studies [8]. We assessed PCSK-9 binding to the two major phenolic acids in the aqueous fraction to better understand the mechanism. This protein regulates LDL receptor expression; thus, it is a target of many cholesterol-lowering agents [37]. Therefore, protocatechuic acid was found to bind firmly with this protein, suggesting its possible inhibition of PCSK-9 activity and subsequently enhanced LDL receptor expression. This mechanism was also suggested by several authors who have worked on the lipid-lowering effect of natural substances [38,39].

Furthermore, many epidemiological and experimental studies reported that the HCD resulted in several interrelated metabolic disturbances, including overweight, abdominal fat accumulation, hypertriglyceridemia, hyperglycemia, insulin resistance, and NAFLD [15]. In this study and as mentioned above, most of these disturbances were observed in mice on the HCD alone. However, administration of AAPE and its aqueous fraction significantly corrected these anomalies. The extracts prevented weight gain and especially abdominal obesity in hyperlipidemic mice. Since hypertriglyceridemia and abdominal obesity are connected, the two extracts’ lipid-lowering action is likely responsible [40].

Additionally, these extracts inhibit liver triglyceride accumulation, which can cause hepatic steatosis, insulin resistance, and hyperglycemia. Treatment reduces plasma glucose and improves liver morphology and histology in mice. Other research suggests liver and plasma energy metabolism causes these effects [41,42]. So, according to molecular docking results, gallic and protocatechuic acids bind strongly to the lipogenesis enzyme FAS and may potentially inhibit it, as previously reported [43]. These molecules also exhibited high binding energies to the LPL enzyme, which catabolizes triglycerides in plasma, suggesting that they probably activate this enzyme and thus explaining the hypotriglyceridemic activity observed in treated mice compared with controls. Our suggestions align with those reported in several recent studies on the same subject [44,45]. In addition, it has been reported that PPARα nuclear receptors are intensely involved in the regulation of β-oxidation, thereby reducing plasma triglyceride levels and their accumulation in the liver and adipose tissue [46]. In this context, the two phenolic acids identified in the active aqueous phase interact strongly with these receptors, which could probably involve them as PPARα activators and thus explain their hypotriglyceridemic and anti-lipogenic effect. On the other hand, these phenolic compounds have a high affinity for PPARγ receptors, which are known to activate TG catabolism by activating LPL in adipose tissue and increasing insulin sensitivity [47]. This could explain the involvement of Argan pulp phenolic acids in regulating glycemia in treated mice.

The coexistence of hyperlipidemia and oxidative stress has long been considered to be an amplifying factor in fatty liver disease [31,48]. The liver’s accumulated lipids can undergo peroxidation and produce harmful molecules, such as lipid free radicals, that can readily penetrate the cell membrane, leading to tissue damage [49]. In this study, we observed that the HCD significantly disrupts antioxidant defense by reducing the level of glutathione and the activity of antioxidant enzymes (SOD and CAT). This is accompanied by increased transaminase and alkaline phosphatase activities, indicating a state of hepatic steatosis. These observations are consistent with several previous studies demonstrating a strong association between the development of NAFLD and the reduction in GSH, SOD, and CAT levels [16,50,51].

In contrast, treatment with AAPE and its two fractions reinforced antioxidant defense by enhancing hepatic glutathione levels and increasing SOD and CAT activities, resulting in a significant reduction in MDA, which suggests the prevention of oxidative stress in the liver. In addition, these extracts reduced transaminase and alkaline phosphatase activities, showing a significant hepatoprotective effect in treated mice compared with controls. This effect is most likely attributed to the antioxidant effect, especially of the flavonoids contained in Argan fruit pulp, since the ethyl acetate fraction, which is rich in flavonoids, proved to be more active than the aqueous fraction. This was confirmed by the results obtained in vitro, which showed that the flavonoid-rich fraction has an antioxidant and anti-lipoperoxyl radical effect similar to that of BHA, a standard synthetic antioxidant. This anti-radical effect could play a key role in preventing the deleterious impact of lipid oxidation in the liver, which contributes to cell damage.

Furthermore, the molecular docking results also show that the flavonoids contained in the active fraction bind strongly to the enzymes SOD, CAT, and CYP2E1. The cytochrome P450 enzyme CYP2E1 is significantly linked to NALFD by oxidizing free fatty acids and producing too many free radicals [49]. These flavonoids and phenolic acids may prevent NALFD by activating antioxidant enzymes and inhibiting CYP2E1. Several animal models have indicated that medicinal plant flavonoids reduce oxidative stress, transaminase activity, and NAFLD [52,53]. Finally, Argan pulp extract polyphenols have a statistically similar effect to fenofibrate, a standard lipid-lowering drug that lowers plasma TGs, LDL-c, and Glu through several mechanisms, most of which have been reviewed above [54].

On the other hand, the phenolic composition of the AAPE and its fractions revealed significant variations in polyphenol, flavonoid, and tannin content. The ethyl acetate fraction was notably rich in flavonoids, particularly epicatechin, while the aqueous fraction contained higher levels of phenolic acids, with protocatechuic acid as the predominant compound. These findings align with the work of Azizi et al. [12], who also reported high polyphenol content in Argan tree extracts, particularly flavonoids and phenolic acids, contributing to their antioxidant properties. Moreover, El Manfalouti et al. [13] identified a diverse range of phenolic compounds in Argan pulp, including catechin, epicatechin, rutin, and quercetin, which were also detected in the present study. Their findings further highlight the potent antioxidant properties of Argan pulp polyphenols, reinforcing the hypothesis that these compounds may contribute to alleviating lipid metabolism disorders. The separation of these compounds into distinct fractions in the current study enables a more targeted exploration of their bioactive properties, particularly regarding their potential influence on lipid metabolism and oxidative stress.

## 5. Conclusions

This study highlights that Argan fruit pulp, traditionally used in Moroccan medicine, is abundant in phenolic phytochemicals. These compounds demonstrate a significant potential to mitigate lipid metabolism disorders, oxidative stress, fatty liver disease, and related cardiovascular issues. We also note that the crude extract used in traditional medicine seems to have a dual function on lipid metabolism and oxidative stress compared with the fractions taken separately.

## Figures and Tables

**Table 1:** Phenolic content of the Argan fruit pulp extract and its fractions.

	Yield (%)	Total Phenols (mg g^−1^)	Flavonoids (mg g^−1^)	Tannins (mg g^−1^)
AAPE	21%	403.12 ± 2.22	220.65 ± 2.37	90.71 ± 1.96
AF	69%	147.18 ± 1.36 ^a^	18.04 ± 1.11 ^a^	40.33 ± 1.54 ^a^
EF	26%	232.94 ± 1.13 ^ab^	195.88 ± 1.54 ^ab^	5.41 ± 1.03 ^ab^

AAPE: aqueous Argan pulp extract; AF: aqueous fraction; EF: ethyl acetate fraction. ^a^ *p* < 0.001 fractions vs. AAPE; ^b^ *p* < 0.001 EF vs. AF.

**Table 2:** Individual phenolic compounds content in the AAPE and its fractions analyzed by HPLC.

Phenolics	mg g^−1^							
	Gallic Acid	Protocatechuic Acid	Catechin	Epicatechin	Rutin	Hyperoside	Quercetin	Luteolin
RT (min)	1.43	2.66	6.06	9.36	12.27	15.33	21.72	23.06
AAPE	23.17	62.65	19.20	86.10	7.21	77.18	12.30	4.41
AF	21.98	59.19	ND	ND	ND	ND	ND	ND
EF	ND	ND	15.45	82.36	5.12	73.44	8.29	3.58

RT: Retention time, AAPE: aqueous Argan pulp extract; AF: aqueous fraction; EF: ethyl acetate fraction, ND: not detected.

**Table 3:** Binding interactions of Argan pulp polyphenols with key proteins involved in regulating lipid metabolism and oxidative stress.

	PCA	GA	CA	ECA	RUT	HYP	QUE	LUT
ABCA-1	−9.67	−9.51	−6.52	−6.46	−5.98	−6.13	−6.89	−7.01
LXR	−10.15	−9.43	−6.71	−6.66	−6.22	−6.70	−5.40	−6.70
CYP7A1	−8.98	−9.91	−6.54	−5.07	−6.17	−5.89	−6.70	−6.14
FXR	−7.88	−8.89	−6.33	−6.13	−6.55	−5.41	−7.08	−5.80
HMG-CoA-R	−9.43	−8.92	−7.15	−7.06	−7.13	−6.60	−7.02	−6.89
PCSK-9	−10.11	−9.89	−6.42	−6.96	−7.10	−5.90	−6.50	−6.33
LPL	−8.69	−9.75	−5.90	−6.11	−5.33	−7.02	−7.11	−7.20
FAS	−7.92	−8.31	−6.72	−5.43	−7.14	−6.93	−6.89	−6.47
PPARα	−9.55	−8.99	−6.66	−4.22	−6.71	−6.55	−6.44	−6.80
PPARγ	−9.33	−8.86	−6.81	−5.66	−6.81	−6.14	−5.99	−5.47
SOD2	−7.10	−7.23	−8.98	−9.56	−8.51	−8.11	−7.22	−8.12
CAT	−7.88	−6.92	−8.02	−7.81	−8.63	−9.34	−8.14	−8.33

The binding forces are expressed in kcal mol^−1^. PCA, proticatechuic acid; GA, gallic acid; CA, catechin; ECA, epicatechin; RUT, rutin; HYP, hyporoside; QUE, quercetin; LUT, luteolin; ABCA-1, ATP-bending cassette class 1; LXR, liver X receptor; CYP7A1, cholesterol 7-alpha hydroxylase; FXR, Farnesoid X receptor; HMG-CoA reductase, hydroxymethyl glutaryl coenzyme A reductase; PCSK-9, proprotein convertase subtilisin/kexin type 9; LPL, lipoprotein lipase; FAS, fatty acid synthase; PPAR, peroxisome proliferator activating receptor, SOD, superoxide dismutase; CAT, catalase; CYP2E1, cytochrome P450 2E1.

## Data Availability

Data are included within the article.

## Abbreviations

The abbreviations used in this manuscript are as follows:

ABCA1	ATP-binding cassette A1
AF	Aqueous fraction
BHA	Butylated hydroxyanisole
CVD	Cardiovascular disease
Cyp7A1	Cytochrome P450. Family 7. subfamily a. polypeptide 1
EF	Ethyl acetate fraction
FAS	Fatty acid synthase
FEG	Fenofibrate-treated group
FXR	Farnesoid X receptor
HCG	Hyperlipidemic control group
HDL-c	High-density lipoprotein cholesterol
HCD	High-calorie diet
HMG-CoA-R	β-hydroxy β-methylglutaryl-CoA reductase
HPLC-DAD	High-performance liquid chromatography–diode array detection
LDL-c	Low-density lipoprotein cholesterol
LPL	Lipoprotein lipase
LXR	Liver X receptor
MDA	Malondialdehyde
NAFLD	Non-alcoholic fatty liver disease
QUE	Quercetin
HYP	Hyporoside
RUT	Rutin
ECA	Epicatechin
CA	Catechin
GA	Gallic acid
Glu	Glucose
PCA	Protocatechuic acid
CYP2E1	Cytochrome P450 2E1
CAT	Catalase
PCSK-9	Proprotein convertase subtilisin/kexin type 9
EATG	Ethyl acetate fraction-treated group
AFTG	Aqueous fraction-treated group
TG	Triglyceride
TCHO	Total cholesterol
SOD	Superoxide dismutase
PVP	Polyvinylpyrrolidone
RCT	Reverse cholesterol transport
AAPEG	Aqueous Argan pulp extract-treated group
PPAR	Peroxisome proliferator-activated receptor
NCG	Normal control group
